# Supplementary material for: Mussel-Inspired Surface Functionalization of Porous Albumin Cryogels Supporting Synergistic Antibacterial/Antioxidant Activity and Bone-Like Apatite Formation
Source: Gels. 2022 Oct 20;8(10):679. doi: 10.3390/gels8100679 (PMC9602075; doi:10.3390/gels8100679)
Supplement: Supplementary file 1 [file gels-08-00679-s001.zip › ESI.pdf]

Mussel-Inspired Surface Functionalization of Porous Albumin Cryogels Supporting Synergistic Antibacterial/Antioxidant Activity and Bone-Like Apatite Formation

Nabila Mehwish [1], Mengdie Xu [1], Muhammad Zaeem [2] and Bae Hoon Lee [1,3,*]

[1] Engineering Research Center of Clinical Functional Materials and Diagnosis & Treatment Devices of Zhejiang Province, Wenzhou Institute, University of Chinese Academy of Sciences, Wenzhou 325011, China
[2] Wenzhou Medical University, School of Pharmaceutical Sciences, Wenzhou 325000, China
[3] Oujiang Laboratory (Zhejiang Lab for Regenerative Medicine, Vision and Brain Health), Wenzhou 325000, China
* Correspondence: bhlee@wiucas.ac.cn

Supplementary Materials:

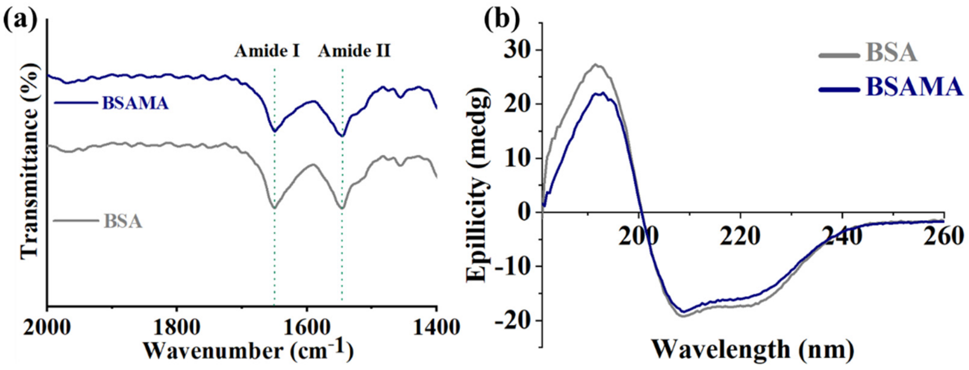

Figure S1. FTIR and CD spectra of BSA and BSAMA.

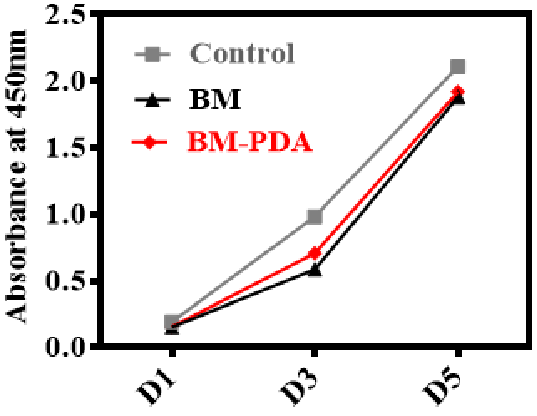

Figure S2. L929 fibroblasts cytocompatibility of BM and BM-PDA cryogels after cultivation for 1, 3, and 5 days.
