# Supplementary material for: Mussel-Inspired Surface Functionalization of Porous Albumin Cryogels Supporting Synergistic Antibacterial/Antioxidant Activity and Bone-Like Apatite Formation
Source: Gels. 2022 Oct 20;8(10):679. doi: 10.3390/gels8100679 (PMC9602075; doi:10.3390/gels8100679)
Supplement: Supplementary file 1 [file gels-08-00679-s001.zip › Supporting videos.pptx]

## Slide 1
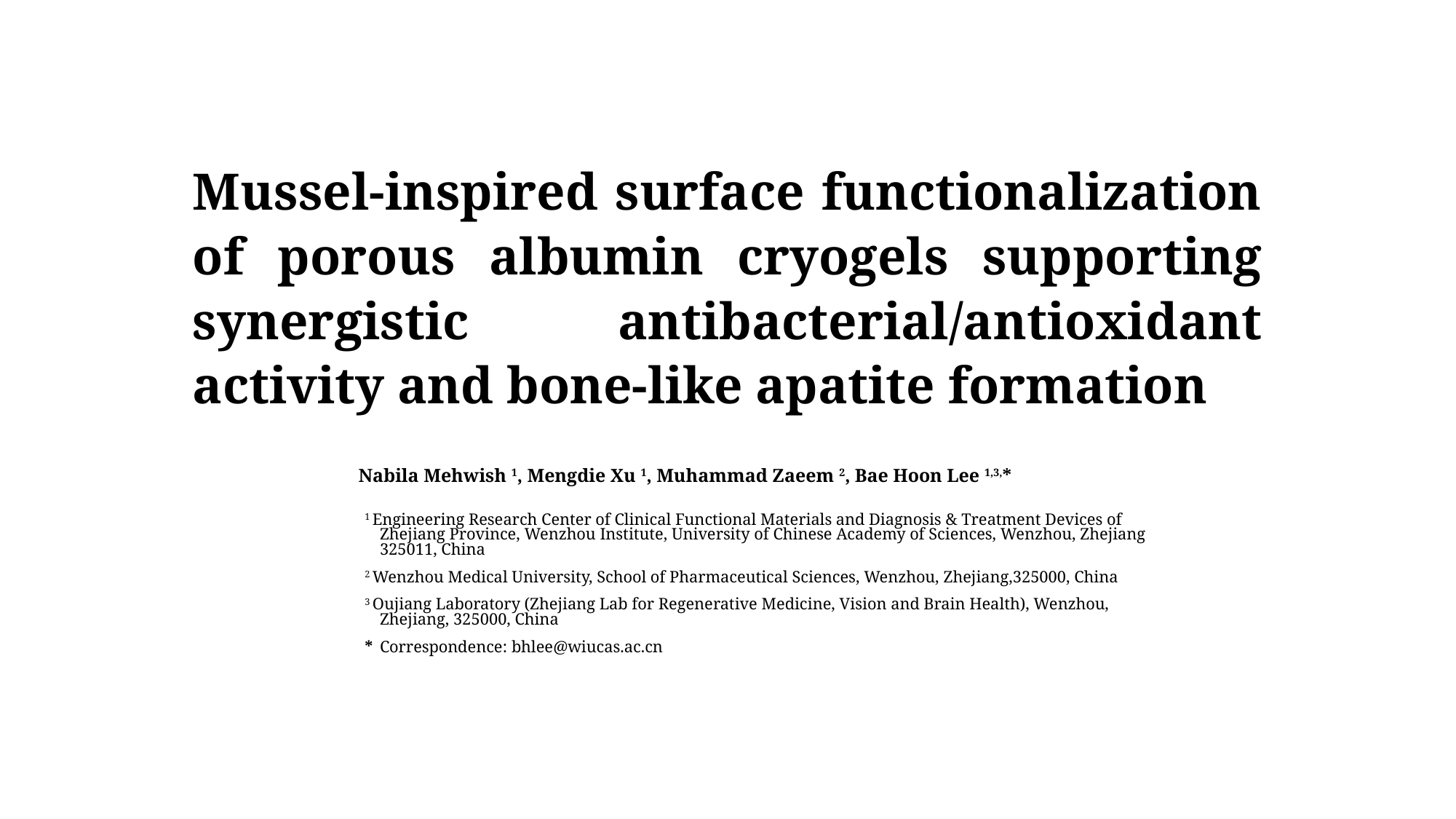

# Mussel-inspired surface functionalization of porous albumin cryogels supporting synergistic antibacterial/antioxidant activity and bone-like apatite formation
Nabila Mehwish 1, Mengdie Xu 1, Muhammad Zaeem 2, Bae Hoon Lee 1,3,*
1 Engineering Research Center of Clinical Functional Materials and Diagnosis & Treatment Devices of Zhejiang Province, Wenzhou Institute, University of Chinese Academy of Sciences, Wenzhou, Zhejiang 325011, China
2 Wenzhou Medical University, School of Pharmaceutical Sciences, Wenzhou, Zhejiang,325000, China
3 Oujiang Laboratory (Zhejiang Lab for Regenerative Medicine, Vision and Brain Health), Wenzhou, Zhejiang, 325000, China
*	Correspondence: bhlee@wiucas.ac.cn

## Slide 2
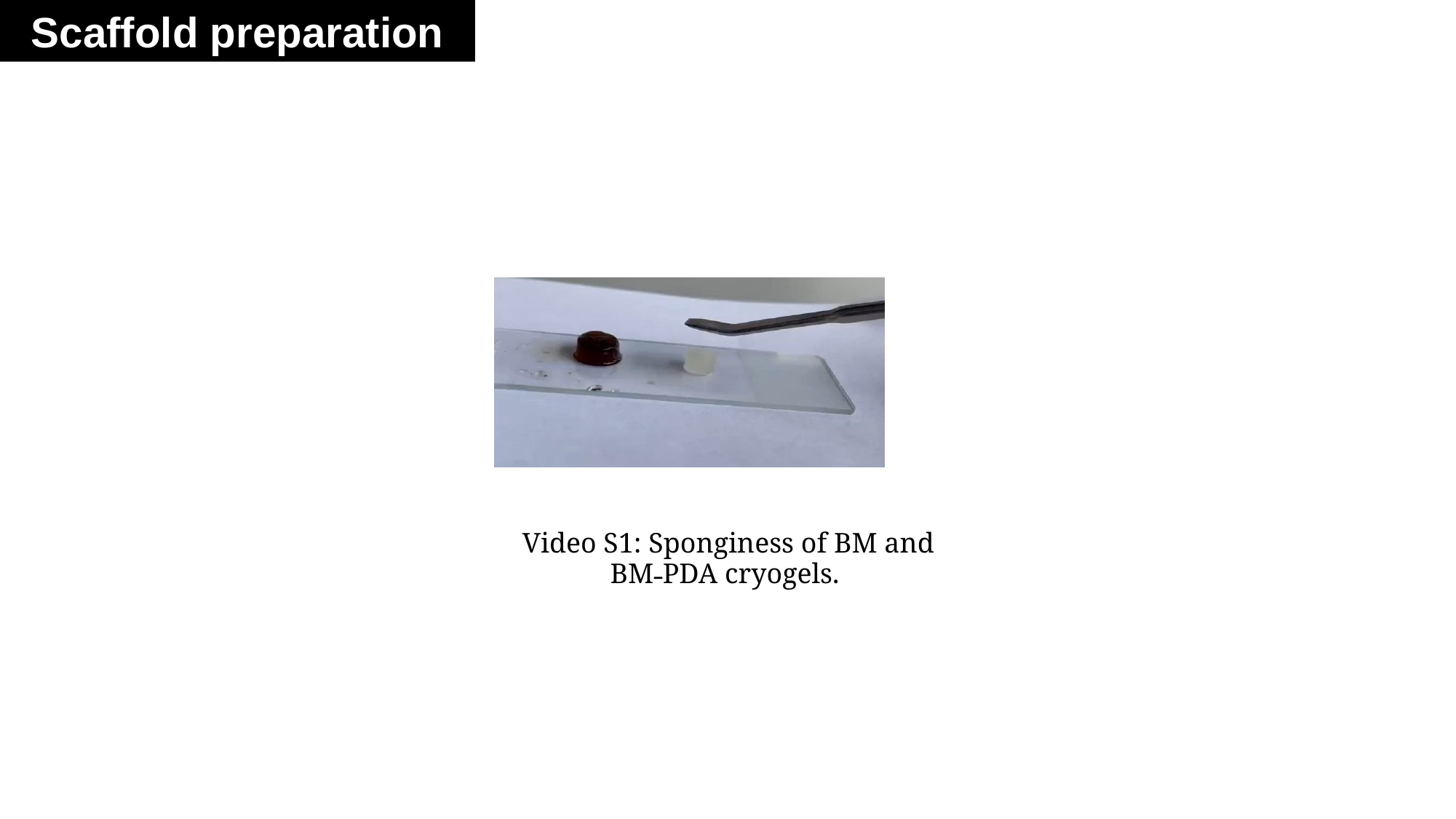

Scaffold preparation
Video S1: Sponginess of BM and BM˗PDA cryogels.

## Slide 3
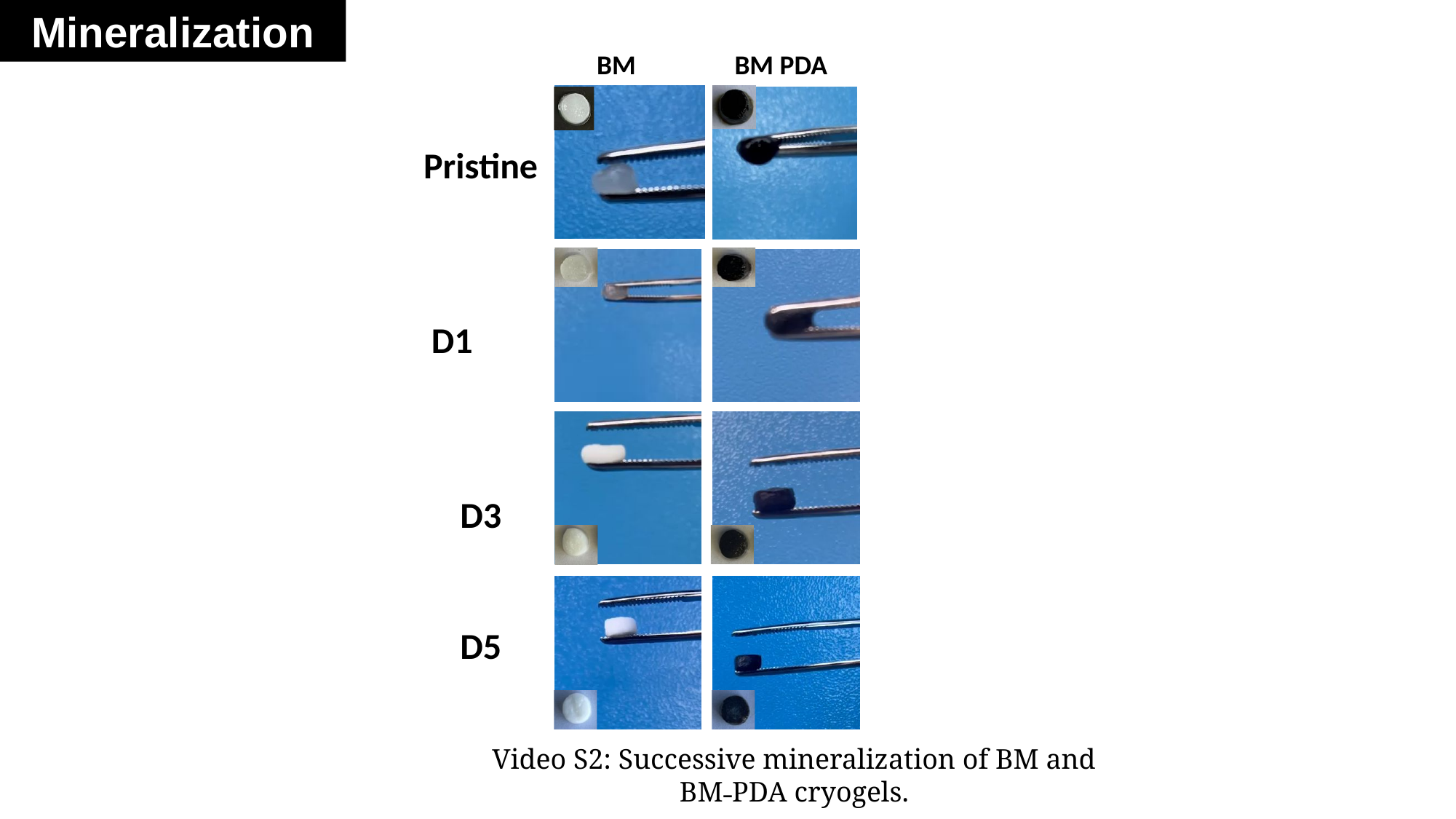

Mineralization
BM BM PDA
Pristine
D1
D3
D5
Video S2: Successive mineralization of BM and BM˗PDA cryogels.

## Slide 4
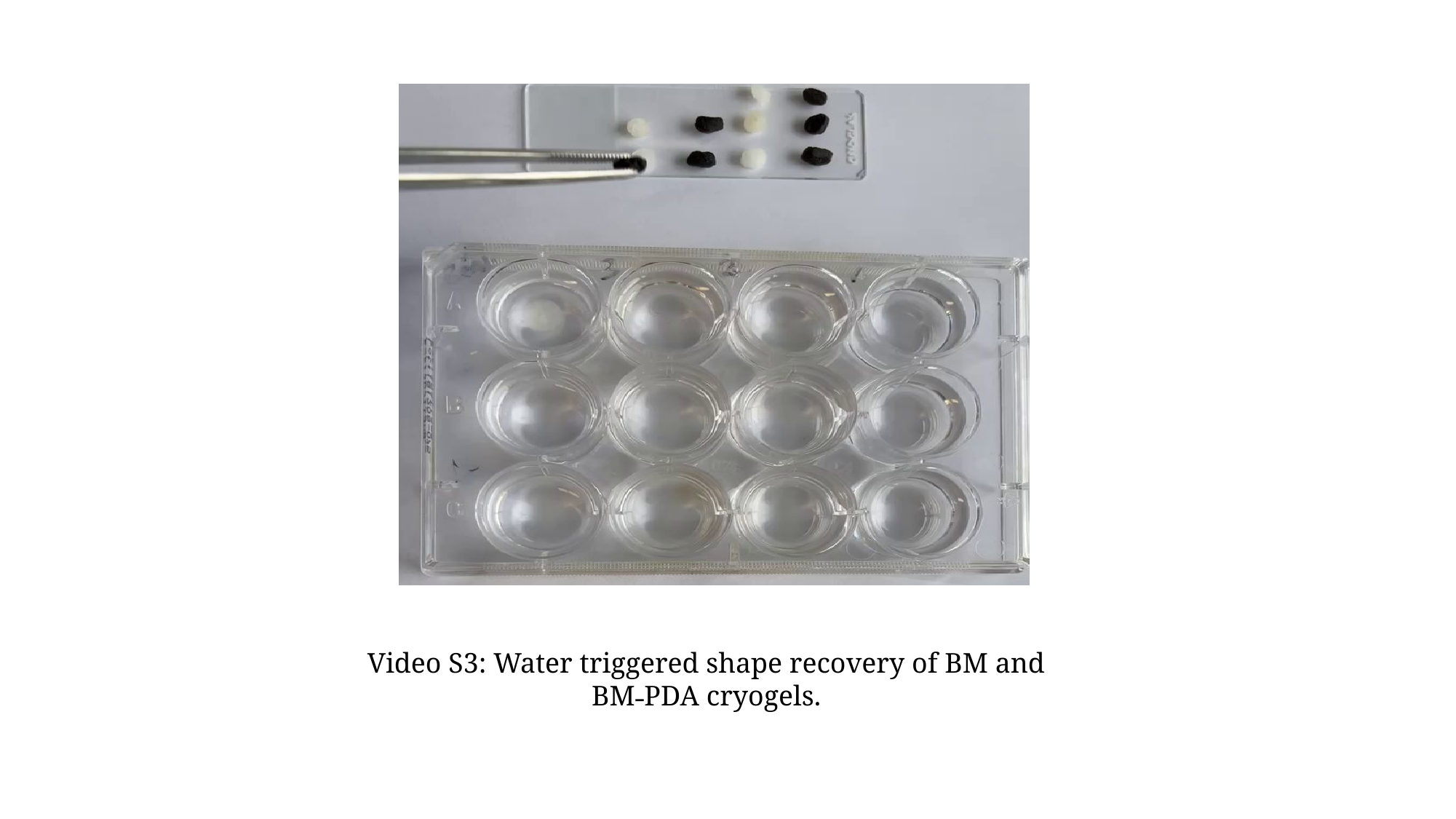

Video S3: Water triggered shape recovery of BM and BM˗PDA cryogels.
